# Symptomatology of irritable bowel syndrome and inflammatory bowel disease during the menstrual cycle

**DOI:** 10.1093/gastro/gov010

**Published:** 2015-03-17

**Authors:** Shishira Bharadwaj, Matthew D. Barber, Lesley A. Graff, Bo Shen

**Affiliations:** ^1^Departments of Gastroenterology/Hepatology, the Cleveland Clinic Foundation, Cleveland, OH, USA; ^2^Department of Obstetrics and Gynecology, the Cleveland Clinic Foundation, Cleveland, OH, USA; ^3^Department of Clinical Health Psychology, University of Manitoba, Winnipeg, Manitoba, Canada

**Keywords:** inflammatory bowel disease, irritable bowel syndrome, menstrual cycle, symptomatology

## Abstract

Gender-related physiological variations in gastrointestinal (GI) symptomatology have been observed in women of reproductive age. Many women experience cyclical changes in GI symptomatology during their menstrual cycle, particularly alteration in their bowel habits. Physiological studies of healthy women during the menstrual cycle showed a prolonged GI transit time during the luteal phase, either in the oro-cecum route or in the colon. Worsened GI symptoms, such as abdominal pain, bloating or diarrhea are observed in patients with irritable bowel syndrome (IBS) during menses. This may be due to elevated prostaglandin levels during menses, with an enhanced perception of viscera-somatic stimuli resulting in nausea, abdominal distension and pain. Also patients with IBS or IBD demonstrate a cyclical pattern more closely related to their bowel habits than healthy controls. Women with inflammatory bowel disease (IBD) also have exacerbated symptoms during menses; however, it is unclear whether this relates to physiological variation or disease exacerbation in IBS or IBD. Studies examining the association of the menstrual cycle and GI symptomatology in patients with IBS or IBD, have not yet clarified the underlying mechanisms. Moreover medications—such as non-steroidal anti-inflammatory drugs and oral contraceptive pills used for dysmenorrhea and menstrual migraine in those patients have not well been controlled for in the previous studies, which can contribute to further bias. Understanding changes in GI symptomatology during the menstrual cycle may help to determine the true extent of disease exacerbation and proper management strategy.

## Introduction

There are gender-related physiological differences in gastrointestinal (GI) symptomatology [1]. Many healthy women experience variations in GI symptoms during the menstrual cycle [2]. The phenomenon may be attributed to the fact that there are sex hormone receptors along the GI tract [3]. In addition, the effect of progesterone on the gut motility during pregnancy—resulting in constipation, increased reflux and biliary dysfunction—suggests a connection between menstrual cycle and the GI symptomatology [4, 5].

GI transit has been extensively studied in healthy women throughout the menstrual cycle; however, the results of both prospective and retrospective studies have been inconclusive. Some studies have shown an increased transit time during the luteal phase [2, 3], whereas others have shown no changes [6, 7]. Possible explanations of the reported discrepancy include differences between study populations, study designs and the markers used to measure GI transit time.

The investigation of an association between GI motility and menstruation in patients with irritable bowel syndrome (IBS) is rather more conclusive [8]. There may be a role of an increased perception of visceral somatic stimuli, resulting in nausea, abdominal distension, and pain, particularly during the first day of menses, probably due to the elevated prostaglandin (PG) levels during menstruation [9, 10]. Recent studies have also shown the variability of GI symptomatology in patients with inflammatory bowel disease (IBD) during the menstrual cycle [11]. There is a general perception that women with IBD experience worsened GI symptoms during menses [11], but there is disagreement over whether this is a physiological exacerbation or a disease flare. In most studies, diarrhea is the most common GI symptom reported during menses, irrespective of the underlying disease. In this article, we discuss the association between the menstrual cycle and GI symptoms in healthy individuals and in women with IBS or IBD.

## Influence of estrogen, progesterone and prostaglandins on motility, inflammation and visceral sensitivity

The menstrual cycle lasts for 28 ± 4 days and is divided into the follicular, luteal and menstrual phases. Estrogen level peaks in the middle of the follicular phase and drops after ovulation, followed by a rise in both estrogen and progesterone levels in the early luteal phase [12]. A sudden drop in both of these hormone levels in the late luteal phase leads to menses. It is generally believed that some healthy women experience perimenstrual alterations in bowel habits [13]. The rate at which food and digestive products transit through the GI tract reflects the integrated activity of intestinal smooth muscle and the influence of factors such as sex hormones [14]. The assumption is that fluctuations in these hormones produce variations in GI symptoms through sex steroid receptors that are present in the GI tract [8].

A number of human and animal studies have evaluated the effects of sex hormones on GI motility [15–17]. The effect of sex steroids on GI motility is more convincing in animals than in humans; in one study, male rats chronically treated with estradiol and progesterone had a slower intestinal transit than controls [15]. Also, a separate study reported an increased gastric emptying during the late luteal phase, in which the estrogen levels were at their lowest [18]. Additionally, another study of the dose-dependent response of progesterone on GI transit reported that a low dose of progesterone retarded intestinal transit, while a high dose reduced the transit time [19]. In addition, colonic transit in rats was shown to be hastened by ovariectomy [20]. In humans, the majority of the studies in healthy women have been inconclusive, with few suggesting an increased GI transit during the luteal phase [16, 17]. There are no published studies comparing gender differences on GI transit in patients with IBS or IBD. At the molecular level, estrogen enhances GI motility through both estrogen receptor-dependent and -independent mechanisms [21, 22]. Estrogen receptor-independent mechanisms include direct activation of K + channels and direct inhibition of voltage-dependent Ca^2+^ channels. On the other hand, progesterone acts via the progesterone receptors regulating intracellular G proteins that mediate smooth muscle relaxation [23].

Estrogen alters pain perception through a number of mechanisms involving the afferent sensory system, opiatergic and serotonergic systems, and stress responses [24–27]. In the afferent sensory system, estrogen modulates sensations of pain through a glutamatergic mechanism or by an increased synthesis of neurotrophins [24, 25]. Also, estrogen enhances opioid receptor-mediated neurotransmission in the thalamus, nucleus accumbens, and amygdala [26]. Additionally, estrogen enhances serotonergic response in the central nervous system (CNS) by increasing synthesis, decreasing uptake and reduced degradation, and by enhancing post-synaptic responsiveness [27]. Estrogen is also known to modulate pain through the autonomic nervous system and hypothalamic-pituitary axis [28, 29]. Studies have shown an increase in sympathetic tone in the mid- to late luteal phase when serum estrogen and progesterone concentrations are at their highest levels [28]. Finally, progesterone and its metabolites have also been found to modulate afferent sensory pathways, which are major inhibitory receptors in the CNS [30], through their effect on the γ-aminobutyric acidergic system. 

Ovarian hormones influence visceral sensitivity and inflammation through serotonergic pathways, mast cell regulation and modulation in stress response. During late luteal phase, decreased serum estrogen and progesterone levels are associated with increased colonic 5-hydroxytryptamine-3 (5-HT) receptor expression, leading to increased GI symptomatology and visceral sensitivity [31]. Additionally, estrogen may cause mast cell degranulation, with release of inflammatory mediators and an increase in visceral sensitivity [32]. Further, estrogen modulates cortisol receptors within the enteric neuron during stress response, resulting in increased visceral sensitivity [33]. In addition, ovarian hormones modulate visceral sensitivity by activation or inhibition of NK1 receptors in the colon [34]. Finally, estrogen and progesterone can produce both proceptive and antinociceptive effects, depending on the pathways being modulated, their serum concentrations, phase of menstrual cycle, and their net effect.

The onset of menstruation is associated with an increase in uterine PGs, particularly PGF2α and prostacyclin, which have a powerful stimulatory effect on the motor activity of the gut [35]. It is unclear whether PGs released from the uterine muscle gain access to the systemic circulation or whether corresponding changes in intestinal PGs cause these changes. A higher frequency of defecation during the menstrual phase is also thought to be related to the release of PGs [36]. Also, PGs and their metabolites are implicated in the inflammatory process in patients with IBD [37]. In contrast, there is speculation that estrogen has anti-inflammatory properties. Estrogen receptors are expressed in the CD4+ and CD8+ T cells, B cells, monocytes, and macrophages. Those receptors are involved in the inhibition of leukocyte recruitment, the increase of epithelial cell proliferation, and a decrease in apoptosis and cellular adhesion molecules [38, 39].

## The menstrual cycle and gi symptoms in healthy women

There have been a number of studies in humans which investigated the effects of the different menstrual cycle phases on small and large bowel motility [1–3, 6, 7, 16, 17, 40–42] (Table 1). These studies have included orocecal, colonic and whole gut transit [3, 16, 17]. Wald *et al.* studied GI transit time using the hydrogen breath test during the follicular and luteal phases of the menstrual cycle and found significantly longer transit times in the luteal phase than in the follicular phase [3]. The authors interpreted the phenomenon as being related to the effects of progesterone. This notion was supported by a study by Davies *et al.*, which reported that women in the luteal phase had a significantly longer gut transit time [16]. The authors further reported that the mean colonic transit time was inversely proportional to dietary fiber intake; thus the colonic transit time in vegetarians was shorter than that in omnivores and dietary fiber supplements were effective in reducing it. Jung *et al.* also showed an increased colonic transit time during the luteal phase [17]; however, several other studies reported minimal effects of the menstrual cycle on orocecal, colonic, or whole gut transit [40, 42]. Possible explanations for the discrepancy in the results could be the methods used to measure GI transit, which included the hydrogen breath test, scintigraphy, and radio-opaque markers, sample size, heterogeneity of patient populations and non-validation of the cycle phase.

**Table 1.** Published studies of normal GI transit during the various phases of the menstrual cycle

Study	Sample size	Age (range in years)	Bowel studied	Phases of menstrual cycle	Methods used for GI transit	Findings
Rees and Rhodes [1] 1976	67	18–45	NA	During menses	Questionnaire	64% had change in bowel habits
Wald *et al.* [3] 1981	15	24–39	Stomach to ileo-cecal	Follicular and luteal phases	Hydrogen breath test	Increased transit time during the luteal phase
Davies *et al.* [16] 1986	51	5–71	Gut	7 on OCPs; 6 in luteal phase; 17 in other phases and post-menopausal	Radiopaque	Increased transit time during the luteal phase
Simmons *et al.* [2] 1988	7	25–44	Gastric	Menstrual cycle	Fasting and response to liquid bolus	Intra-gastric recordings indicated greater changes during menses than mid-cycle
Kamm *et al.* [6] 1989	18	22–47	Gut	Follicular and luteal phases	Radiopaque	Hormones have no effect on GI transit
Turnbull *et al.* [40] 1989	35	NA	Gut	Menstrual cycle	Oro-cecal transit: hydrogen breath test Gut transit: radiopaque	Hormones have no effect on GI transit
Hinds *et al.* [7] 1989	36	NA	Colonic	Pre- and during menses	Radiopaque	Hormones have no effect on GI transit
Bovo *et al.* [41] 1992	20	24–32	Oro-cecal	NA	Hydrogen breath test	Hormones have no effect on GI transit
Degen *et al.* [42] 1996	32	19–45	Colonic	NA	Scintigraphy	Hormones have no effect on GI transit
Jung *et al.* [17] 2003	40	58–60	Colonic	NA	Radiopaque	Increased colonic transit during luteal phase

GI = gastrointestinal; NA = not available; OCP = oral contraceptive pills

In summary, physiological studies of gut transit time throughout the menstrual cycle were inconclusive, although a few of them indicated prolonged transit times during the luteal phase, either in the small bowel or the recto-sigmoid colon.

## The menstrual cycle and ibs

IBS is a functional bowel disorder defined by symptom-based diagnostic criteria in the absence of detectable organic or structural causes. The prevalence of IBS in the general populations of Europe and North America is estimated to be 10–15% [43]. There is an overall female predominance with a 2:1 ratio [44]. In the absence of biological markers, efforts have been made to standardize the diagnosis of IBS using symptom-based criteria (the Manning- and Rome Criteria) [45]; the Rome Criteria have undergone several revisions and are currently used in clinical practice. In addition to symptoms of abdominal discomfort, changes in bowel habit and bloating, patients with IBS often experience a broad range of non-GI symptoms, including impaired sexual function, dysmenorrhea, dyspareunia, increased urinary frequency and urgency, and body pain [45]. IBS is classified into subtypes with constipation (IBS-C) or diarrhea (IBS-D) predominant, and an alternating or mixed pattern (IBS-M) [46]. The etiopathogenesis of IBS is multifactorial; no single treatment is currently regarded as being universally applicable to the management of all IBS patients [46]. Due to the common association between IBS symptoms and factors such as diet, stress, and psychological factors, psychological and behavioral interventions are also recommended to alleviate, if not eliminate, such exacerbating factors [47].

The predominance of IBS in females suggests that sex hormones play a role in the disease process [44]. Women with IBS have an increased perception of somatic and visceral sensitivity, linked temporally to a dynamic decrease in the levels of ovarian hormones during menses [48]. Also rectal sensitivity—which involves self-reported discomfort, urgency, and desire to defecate in response to balloon insufflation on barostat examination—varies with the menstrual cycle in women with IBS, in contrast to healthy controls [49]. Whitehead *et al.* studied GI complaints associated with menses in women with IBS, and found that those patients were more likely to experience increased flatulence, diarrhea, or constipation during menses than controls [50]. Similarly, Heitkemper *et al.* reported that IBS patients experienced more stomach pain, nausea, and diarrhea during menses than controls [51]. In a retrospective study, Kane *et al.* reported that women with IBS had an increase in abdominal pain, diarrhea or constipation during pre-menses and menses [52]. Many of the studies described have relied on symptom recall, in which women were asked about their experiences of GI symptoms in various phases of their menstrual cycle [8, 49–55] (Table 2). Obviously, this approach might have resulted in recall bias. To mitigate recall bias, other investigators have used daily dairies to prospectively study the pattern of GI symptoms throughout the menstrual cycle [8, 51]. Most studies that involved prospective recordings using a symptoms dairy also found that GI symptomatology was more severe during both menses and pre-menses in IBS patients, further supporting the association between the menstrual cycle and IBS symptoms.

**Table 2.** Published studies on IBS and the menstrual cycle

Study	Study design	No. studied /control	Menstrual phase	Methods	IBS diagnostic criteria	Symptoms	Findings
Whitehead *et al.* [50] 1990	Retrospective	72/234	During menses	Questionnaire	Manning	GI	IBS/FBD symptoms worse during menses
Heitkemper *et al.* [8] 1992	Prospective	19/39	2 menstrual cycles	Diary	Rome I	GI, somatic	IBS/FBD symptoms worse pre- and during menses
Heitkemper *et al.* [51] 1995	Prospective	44/25	1 menstrual cycle	Diary	Rome I	GI	IBS and IBS-NP groups reported more symptoms than the control group during the entire cycle
Kane *et al.* [52] 1998	Retrospective	46/90	Pre- and during menses	Questionnaire	Rome I	GI, somatic	IBS group reported a cyclical pattern to menstrual cycle more often than control
Lee *et al.*[53] 2001	Retrospective	714	1 menstrual cycle	Questionnaire	Rome I	GI, somatic, psychological	No effect of menstrual cycle
Houghton *et al.* [49] 2002	Prospective	29	1 menstrual cycle	Ano-rectal distension/diary	Rome I	Rectal sensitivity, GI, somatic	Abdominal pain and bloating worse during menses
Heitkemper *et al.* [54] 2003	Prospective	149/42	1 menstrual cycle	Diary	Rome I	GI, somatic, psychological	GI symptoms worse pre- and during menses
Lee *et al.* [55] 2007	Cross-sectional	253/252	1 menstrual cycle	Questionnaire	Rome II	GI	No effect of menstrual cycle

FBD = functional bowel disease; GI = gastrointestinal; IBS = irritable bowel syndrome; IBS-NP = irritable bowel syndrome: non patient

Gonadal hormones exert their pain sensitivity effects via a variety of mediators, including 5-HT receptors, which are present throughout the GI tract [56]. Experimental studies and clinical trials have illustrated the role of 5-HT receptors in abdominal pain perception and GI tract motility [57, 58]. As a result, antagonists to 5-HT receptors have been found to be efficacious in the treatment of abdominal pain and discomfort [58].

Due to the small number of longitudinal studies that have compared GI symptoms in pre- and post-menopausal women, there is insufficient evidence to determine the effect of menopausal status on IBS symptoms. Generally, population surveys suggest that women experience a decrease in IBS following menopause [59]; however, many women have also reported an increase in IBS symptoms around the onset of menopause (perimenopause) [60]. It has been speculated that this increase in symptoms early in the menopause is due to decreased levels of sex hormones, in a similar way to the increase in IBS symptoms in the days around onset of menses. Ruigomez *et al.* reported that postmenopausal women who use hormone replacement therapy (HRT) are more likely to develop IBS than women who do not, further supporting the role of sex hormones in IBS [61]. Additionally, Palomba *et al.* found that treatment with a gonadotropin releasing hormone agonist reduces the severity of IBS symptoms [10].

In summary, based on the studies to date, female sex hormones appear to influence the severity of IBS symptoms. This is further substantiated by an observed improvement in IBS symptoms after ovariectomy and with the use of GnRH agonists [60, 61]. IBS is a multicomponent disorder involving alterations in visceral sensitivity, central processing of neuronal signals, autonomic nervous system, and gut motility, and several factors—including sex hormones—play a role. Therapeutic interventions for IBS have not yet incorporated any consideration of sex hormones, but this is a promising avenue for development.

## The menstrual cycle and ibd

The role of the menstrual cycle in the symptomatology of IBD patients is complex and not completely understood. The literature has shown that there are cyclical changes in GI symptoms in IBD patients during the menstrual cycle [55, 62]. The exact etiology of the fluctuations in bowel pattern in relation to ovarian hormones remains to be defined; however, a possible role of PGs has been hypothesized. PGs are important components of inflammation in IBD and they are released by the endometrium during menses, which can exacerbate GI symptoms [62]. To date, only a few studies have investigated the relationship between GI symptoms in IBD and the menstrual cycle [11, 52, 62, 63] (Table 3); according to these, the majority of IBD patients reported worsening of GI symptoms during premenstrual and menstrual phases [11]. Most of these studies involved questionnaires and small sample sizes, with large inter-individual variability. Despite these limitations, it appears that the IBD patients experienced worsening of GI symptoms during the premenstrual and menstrual phases.

**Table 3.** Published studies on IBD and the menstrual cycle

Study	Study design	No. studied, UC/CD/control	Age (range in years)	Menstrual phase	Methods	Findings
Kane *et al.* [52] 1998	Retrospective	49/49/90	20–48	Pre- and during menses	Questionnaire	1) IBD group reported a cyclical pattern to menstrual cycle more often than controls
						2) Diarrhea: most common symptom reported
Parlak *et al.* [62] 2003	Prospective	38/21/38	30–47	Menstrual cycle	Diary	1) UC and control groups reported more GI symptoms during menses
						2) CD symptoms worse pre-, and during menses compared to postmenses
Bernstein *et al.* [63] 2012	Retrospective	87/151/156	18–65	Pre-, and during menses	Questionnaire	1) CD and UC groups more likely to experience diarrhea during menses
						2) Premenstrual phase- CD patients more likely to report worsening of their IBD symptoms than UC and control
Lim *et al.* [11] 2013	Prospective	27/13/44	33–43	Menstrual cycle	Questionnaire	GI symptomatology worse during menses in IBD and control groups

CD = Crohn’s disease; IBD = inflammatory bowel disease; UC = ulcerative colitis

The effect of menopause on IBD disease activity is unclear. In 1989, Lichtarowicz *et al.* surveyed women with Crohn’s disease (CD) for details of their menstrual cycles, ages at menopause and smoking habits [64]. Of the 146 patients with CD who responded, 48 (34%) had undergone menopause at a mean age of 47.6 years, compared with 49.6 years in the control group. The investigators concluded that CD was associated with premature menopause [58]. In contrast, a retrospective study by Kane *et al.* reported no difference between the IBD and control groups in the age of onset of menopause [65]. Additionally, there was no significant correlation between having a flare in the pre- and post-menopausal states; however, the use of HRT appeared to confer a protective effect in on disease activity [65]. Compared with those not using HRT, women with IBD who used HRT were 82% less likely to have a flare in the first 2 years of menopause. Moreover, those on HRT who did have a flare appeared to have a less-severe disease course [53].

Limited data are available on the variability of GI symptomatology during the menstrual cycle in patients with restorative proctocolectomy with ileal pouch-anal anastomosis (IPAA). One study, which included 123 patients with IPAA, reported increased abdominal pain, diarrhea, and urgency during menses compared with pre-menses. Also, painful menses was significantly associated with increased GI symptomatology in those patients (odds ratio 5.67; 95% confidence interval 1.41–22.88; *P = *0.015) [66].

The association between the use of oral contraceptive pills (OCP) and development of disease activity in IBD has been extensively investigated. Cornish *et al.* summarized data from 14 studies and found that female patients exposed to the OCP have a pooled relative risk (RR) of 1.46 of developing CD with current exposure to the OCP, even after adjusting for smoking [67]. The role of OCPs in the disease activity of IBD is less clear, although limited evidence suggests that there is no greater risk of relapse or increase in disease activity score with the use of OCP [68, 69].

## Clinical implications

Many characteristics of both IBS and IBD have a significant impact on various aspects of a patient’s quality of life; in addition, female patients can be affected by fluctuations in hormones from menarche to menopause [69, 70]. When providing care to women with IBD, physicians should be aware of these gender-specific issues, including symptom fluctuations during the menstrual cycle, body image, sexuality, contraception, cervical cancer screening, fertility and pregnancy and lactation, as well as matters related to the menopause and HRT [70, 71]. Marri *et al.* reported a higher rate of voluntary childlessness in women with IBD than in controls [72]. The use of OCPs to prevent pregnancy is also common in patients with IBD [71]. Furthermore, OCPs are frequently prescribed for patients who have a combination of dysmenorrhea and menstrual migraine [73, 74]. Additionally, due to poor absorption, there is always a risk of OCPs being ineffective in active disease and in patients with multiple surgeries [75]. Osteoporosis following menopause is another concern [76]. The use of HRT in patients with IBD may help reduce risk of flare-up and prevent bone loss but, due to the estrogen effect, there is a small risk of breast cancer, coronary artery disease, stroke, and venous thromboembolism [69].

Dysmenorrhea and menstrual migraine are problems commonly experienced by women of reproductive age [72, 73]. Dysmenorrhea is linked to the excess or imbalance in PG’s and arachidonic acid metabolites released from the endometrium during menstruation, leading to uterine cramping and pain. In addition, these may gain access to the systemic circulation and GI tract, causing abdominal pain and diarrhea. Few studies have investigated the prevalence of dysmenorrhea in patients with IBS and IBD. One, which compared IBS patients with healthy controls, found a higher incidence of dysmenorrhea in patients with IBS [77]. Also another study, which used daily diaries to record symptoms, found a higher pain score in the late luteal and menstrual phases in patients diagnosed with simultaneous IBS and dysmenorrhea than in patients with IBS alone [78]. Similarly, one study found an increased prevalence of dysmenorrhea in patients with CD [79]. In conclusion, dysmenorrhea is highly prevalent in patients with IBS and IBD. Further, in patients with IBS- or IBD comorbid dysmenorrhea, it may be difficult, due to the co-existence of the conditions, to distinguish between an active flare and increased symptom scores during the menstrual phase.

Very few studies have investigated the association between endometriosis, polycystic ovarian disease (PCOS) and IBS or IBD. A questionnaire study comparing 50 women with IBS and 30 with endometriosis showed a considerable overlap of reported symptoms between the two groups [80]. Also, a case-control study of women aged 15–55 years showed that women with endometriosis were more likely to be diagnosed with IBS [81]. The authors of these studies speculated that there was a significant overlap of symptomatology between the two diseases. In patients with IBD, a population study of 37 661 women with endometriosis showed a 50% increase in their risk of IBD [82]; the reason stated was the shared immunological pathogenesis of the two diseases. There is a paucity of data on PCOS with IBS or IBD. A questionnaire study which compared patients with PCOS with healthy controls found an increased prevalence of IBS in patients with PCOS [83]. In conclusion, although few studies have reported increased prevalence of endometriosis and PCOS in patients with IBS or IBD, there could be a component of misclassification bias due to overlap of the symptoms between these groups.

After significant exposure to estrogen at the onset of menses, a decline in estrogen concentration is a trigger for migraine in some women. There has been concern about frequent use of non-steroidal anti-inflammatory drugs (NSAIDs) in patients with dysmenorrhea and migraine and underlying IBD. While a recent review did not support NSAIDs as an etiological agent in IBD, a weak association between use of NSAIDs and IBD flares was reported [84].

Recent research has focused on the influences of stress and psychological health on disease activity in patients with IBS and IBD. A large, national, population-based study found that individuals with IBD were more likely to report poor general health, while people with IBS were more likely to report poor mental health [85]. Anxiety and depression are common mood symptoms experienced by both IBS and IBD patients [86]. Hypnosis, biofeedback and psychotherapy help to reduce anxiety [87]. A recent meta-analysis demonstrated a significant benefit from antidepressants in patients with IBS, which was less in those with IBD [88]. Behavioral intervention was able to prolong remission in IBD patients more than a year [89]. The general effects of psychotherapy on the disease course of IBD have been mixed, but are nevertheless promising [90]. The value of behavioral and psychological interventions for women with IBD during different phases of menstrual cycle has not been studied. Ideally, there is a need for a multi-specialty approach to the care of women with IBD, which includes gastroenterologists, gynecologists and psychologists.

## Summary and conclusion

Based on this review, we conclude that hormone fluctuations in women of reproductive age influence the GI function, particularly in patients with IBS or IBD. There are several common events in women’s lives that result in dramatic changes in sex hormone levels, including the use of OCPs, pregnancy, lactation, surgical removal of the ovaries, and HRT. Additionally, gonadal hormones influence pain sensitivity via a variety of mediators including serotonin, which is ubiquitous in the GI tract and causes smooth muscle hypersensitivity associated with IBS. Notably, few studies have observed increased GI transit time during the luteal phase; however, these studies have been limited by sample size, study design, differences between the GI markers used, and wide inter-individual variability.

Research on the effects of ovarian hormones on IBS and IBD has been more conclusive. Premenstrual and menstrual worsening of GI symptoms has been reported more frequently by patients with IBS or IBD than by controls. Additionally, both disease groups report a cyclical pattern to their bowel habits. Knowledge of cyclical changes in GI symptoms during the menstrual cycle may help us quantify the true exacerbation. A carefully recorded menstrual history may be helpful before considering major changes in therapeutic intervention in patients with IBD or IBS. In women with IBS or IBD, in addition to a good menstrual history, clinicians should give a special consideration to gender-related issues such as contraception, cervical screening and HRT. Further, osteoporosis is a problem that must be considered in patients with IBD, irrespective of their postmenopausal state.

Since most studies have limitations—including retrospective design, small sample sizes, non-standardized self-report of symptoms and large inter-individual variability—future avenues of research should include prospective recording of symptoms with respect to different phases of the menstrual cycle. Also, the phase of the menstrual cycle should be validated by serum- or urine analysis of sex hormones. Further, simultaneous measurement of inflammatory markers during symptom flare-up would help to distinguish between acute exacerbation and normal physiological response.

An important next step is to determine whether sex or hormone status affects the efficacy of current therapeutic approaches. Additionally, medications such as NSAID and oral contraceptives used for dysmenorrhea and menstrual migraine have rarely been controlled for, in the previous studies, which might have contributed to further bias. Categorization and selection of patients on IBS subtypes may also be needed. Furthermore, the value of sex hormones (e.g. OCP and HRT) in the management of patients with IBS or IBD remains to be clearly defined. Finally, the role of behavioral and psychological interventions for women with IBS or IBD during different phases of menstrual cycle could also be an area of further research.

*Conflict of interest statement*: none declared.
